# Intention-Based Critical Factors Affecting Willingness to Adopt Novel Coronavirus Prevention in Pakistan: Implications for Future Pandemics

**DOI:** 10.3390/ijerph18116167

**Published:** 2021-06-07

**Authors:** Munir Ahmad, Nadeem Akhtar, Gul Jabeen, Muhammad Irfan, Muhammad Khalid Anser, Haitao Wu, Cem Işık

**Affiliations:** 1 School of Economics, Zhejiang University, Hangzhou 310058, China; munirahmad@zju.edu.cn; 2 School of Urban Culture, Nanhai Campus, South China Normal University, Foshan 528225, China; 3 Pakistan Center, North Minzu University, Yinchuan 750001, China; 4 Research Institute of Business Analytics and Supply Chain Management, College of Management, Shenzhen University, Shenzhen 518060, China; guljabeen@ncepu.edu.cn; 5 School of Economics and Management, North China Electric Power University, Beijing 102206, China; 6 School of Management and Economics, Beijing Institute of Technology, Beijing 100081, China; irfansahar@bit.edu.cn (M.I.); haitao.kungfuer@gmail.com (H.W.); 7 Center for Energy and Environmental Policy Research, Beijing Institute of Technology, Beijing 100081, China; 8 School of Public Administration, Xi’an University of Architecture and Technology, Xi’an 710000, China; mkhalidrao@xauat.edu.cn; 9 Faculty of Tourism, Anadolu University, 26470 Tepebaşı-Eskişehir, Turkey; cemisik@anadolu.edu.tr

**Keywords:** intention-based critical factors, novel coronavirus, pandemic prevention, COVID-19, hybrid theoretical framework, path modeling, Pakistan

## Abstract

Since human beings have a long tradition of coexistence with pandemics, which may profoundly impact them, adopting preventive measures is crucial for humankind’s survival. This study explores the intention-based critical factors affecting the willingness of individuals to adopt pandemic prevention. To this end, a representative sample of 931 Pakistanis filled in an online questionnaire. However, only 828 questionnaires were found to be complete and valid for path modeling analysis. The core findings are as follows: Firstly, peer groups’ beliefs, self-efficacy, perceived risk, pandemic knowledge, ease of pandemic prevention adoption, and risk-averse behavior are revealed as driving forces of the individuals’ willingness to adopt pandemic prevention. Contrastingly, a lack of trust in political will and mythical attitude towards pandemics are uncovered as inhibitors. Nevertheless, moral values depict a neutral role. Secondly, the peer groups’ beliefs are highest ranked, followed by the lack of trust in political will and a mythical attitude towards pandemic prevention. Finally, moral values are determined as the lowest-ranked critical factor. Based on these results, the government should promote awareness campaigns on lethality and fatality of the pandemic at both centralized and decentralized levels to win people’s trust at the grass-roots level and overcome the mythical attitude of individuals at all societal levels. Besides, access to personal protective gears should be made feasible since an easier pandemic prevention adoption would increase the individuals’ willingness to adopt such preventative measures.

## 1. Introduction

Since human beings have a long tradition of coexistence with pandemics, which may profoundly impact them, adopting preventive measures is crucial for humankind’s survival. Global pandemics are rising every day because the proper diagnosis of the right people at the right time is missing [1]. The involvement of vaccine producers, health authorities, and governments is essential for monitoring and preventing such pandemics [2].

The Coronavirus Disease 2019 (COVID-19) began in Wuhan (a Chinese city) in late December 2019. In the face of people’s domestic and international mobility, the epidemic eventually turned into a worldwide pandemic. The Chinese government took strict steps to effectively curtail the epidemic outbreak [3]. As of 29 May 2021, an estimated over 169 million cases tested positive, while about 3.5 million patients lost their lives worldwide due to COVID-19 infection. The epicenter of the COVID-19 shifted from Wuhan through Iran and Italy to the United States. The U.S., with more than 33 Million confirmed cases, is the pandemic’s current epicenter, followed by India with more than 27 million cases. Besides, Brazil, France, and Turkey are also among the hotspots of COVID-19 patients, with more than 16, 5.5, and 5.2 million confirmed cases, respectively [4]. Its outbreak started in Pakistan in the middle of March 2020 and reached a peak number of confirmed cases by mid-June 2020. Afterward, the number of cases reduced substantially; however, a resurgence of patients started in the last quarter of October 2020 due to the lack of prevention measures at an individual scale. As of 29 May 2021, around 913,784 cases were reported, whereas the total death toll reached 20,607. In the meantime, an estimated 835 thousand individuals have recovered, which is indeed an optimistic side of the gloomy picture.

To curtail the COVID-19 outbreak, several countries such as Italy, Spain, India, Russia, and China implemented nationwide lockdowns. However, the Pakistani government’s COVID-19 containment strategy was not based on complete lockdown across the nation. Instead, smart and targeted lockdowns were imposed on locations with agglomerated patients [4]. In light of this, the individuals’ willingness to adopt pandemic prevention (WAPP) becomes vital. Consequently, during a pandemic like COVID-19, the individuals’ WAPP is explicitly defined by their intention-based critical factors (ICFs). The ICFs include the driving and inhibitory factors shaping the individuals’ intention to accept or reject pandemic prevention. Since the individuals’ intention performs a critical role in actual behavior [5], the analysis of ICFs would be imperative to understand the COVID-19 prevention measures.

The COVID-19 pandemic has become a hotly debated issue among global scholars; nevertheless, studies on ICFs affecting individuals’ WAPP are scarce. In particular, no research has been identified examining the ICFs involving driving forces and inhibitors of individuals’ WAPP in a hybrid theoretical framework. The previous studies were fundamentally based on the following debates: The first debate comprised the epidemiological characteristics of the epidemic, including “acquired immunodeficiency syndrome” (AIDS), dengue fever, malarial infection, and coronavirus infection [6,7]. The second debate considered the prevention and control of pandemics such as SARS-CoV 2002, MERS-CoV 2012, and COVID-19, belonging to the coronavirus family [8,9,10]. Simultaneously, some studies addressed epidemic prevention and control from the government’s perspective [11,12]. The third debate focused on the links of COVID-19 with the sustainable supply chain [13,14] and environmental features such as humidity and temperature [15,16].

The fourth debate was based on the psychological factors interacting with COVID-19 related attributes, including the intention of being vaccinated, individuals’ resilience, individual susceptibility to conspiracies, prosocial behavior, socio-political predictors, dark personality traits, and psychological entitlement, among others. In this regard, Karataş and Tagay [17] examined and revealed that no experience of trauma, satisfaction of life, and hope were positively linked with adults’ resilience during the COVID-19 outbreak. Karlsson et al. [18] studied and disclosed a positive linkage between the perceived risk of COVID-19 and the intention of being vaccinated in the Finnish context. Hughes and Machan [19] assessed and concluded that Machiavellianism and psychopathy positively influenced COVID-19 related conspiracy beliefs. Jin et al. [20] empirically evaluated and found that the age factor positively impacted individuals’ prosocial COVID-19 response, meaning that older individuals had a relatively higher perceived cost of being infected by the virus. In a different study, Wagerman et al. [21] investigated and revealed that anxious attachment positively determined the COVID-19 distress factor. Hardin et al. [22] analyzed and discovered that Machiavellianism and Narcissism introduced negative impacts in response to COVID-19 in the U.S. context.

Moreover, Zitek and Schlund [23] studied the psychological entitlement in the United States and revealed that the individuals were not concerned about transmitting the disease to others. Therefore, they were less likely to follow the COVID-19 prevention guidelines. Ruggieri et al. [24] investigated pre-and post-quarantine behaviors and found a rise in anxiety, stress, and loneliness, along with a decline in life satisfaction. Chan [25] studied and unveiled that fairness and caring showed compliance with all types of individual behaviors; however, sanctity merely predicted the social distancing and wearing a face mask in the United States. Next, Li et al. [26] studied the community sample in China. They discovered that high perceived risk was linked with increased donations to the COVID-19 patients and the health workers. Paredes et al. [27] examined and found that highly resilient people, who were better at overcoming stressful and traumatic situations, demonstrated relatively less impact of COVID-19 threat on prospective pandemic anxiety and stress. Malesza and Kaczmarek [28] analyzed and concluded that the factors, including a greater amount of protection recommendation, COVID-19 information from diverse sources, and a lack of belief that catching COVID-19 was determined by individuals’ actions, significantly contributed to pandemic-related anxiety.

Besides, Volk et al. [29] investigated and uncovered that the demographic attributes involving income and children were directly linked to COVID-19 handling response. While age, sex, income, and children had an indirect linkage. Grossman et al. [30] studied and disclosed that COVID-19 related concerns were positively correlated with loneliness and sleeplessness. Ahmad et al. [1] studied the influencing factors of the acceptance of COVID-19 protection in China. Their findings showed that guidelines by the Chinese government boosted the epidemic protection adoption in China. However, their study included a highly educated population comprised of government employees. Therefore, the findings of their research cannot be generalized. As a further note, China’s political system is different from that of other democratic nations. Hence, the findings extracted based on their sample cannot be generalized for the other democratic countries. Additionally, no research has been known to introduce the above-stated ICFs to a behavioral framework obtained by integrating the composite of planned behavior (PBST) and reasoned action schools of thought (RAST). Finally, the driving forces and inhibitors of individuals’ WAPP were not previously considered. The understanding of such driving forces and inhibitors would help improve the adoption behavior substantially. Therefore, the investigation of such critical factors is timely and urgent.

To fill the aforementioned gaps, this research investigates the ICFs of individuals’ WAPP in terms of driving forces and inhibitors. From the empirical side, new critical factors involving the lack of trust in political will and mythical attitude towards pandemic are included. Furthermore, a theoretical framework composite of PBST and RAST is integrated to incorporate additional ICFs that determine the WAPP. Those factors include a lack of trust in political will, mythical attitude towards pandemic, perceived risk, pandemic knowledge, the ease of pandemic prevention adoption, risk-averse behavior, and moral values. The empirical outcomes of this work are distinguished from the mainstream literature. The derived policies are equally useful for both the developing and developed nations in the world health emergency during the COVID-19 pandemic as well as potential future pandemics.

The remainder of the study is arranged as follows: Section 2 explains the extraction of a hybrid theoretical framework. Section 3 is based on data, methods, and analysis. Section 4 details the results of this work. Section 5 explains the conclusion and policy suggestions.

## 2. Literature Review and Hypotheses Formulation

### 2.1. Mythical Attitude towards Pandemic

Mythical attitude towards pandemic can be defined as the traditional way of thinking about the existence or non-existence of a pandemic and its influence on human beings. Individuals with mythical attitudes might believe that the pandemic will automatically vanish due to external factors such as high temperature. They might also believe that pandemic prevention is useless for them. In this regard, Latkin et al. [31] studied the linkages of COVID-19 skepticism with protection behavior, social distancing, conspiracy theories, and individuals’ political ideas in the U.S. and revealed the highly skeptical individuals less likely to adopt COVID-19 protection. Alper [32] investigated the correlation between COVID-19 conspiracy theories and protection adoption and revealed no link between the two in the Turkish context. Research was conducted to examine the knowledge, preventive measures, and attitude of live poultry market workers regarding the avian influenza in the Chongqing district of central China by taking a sample of 216 workers of this district. The results exhibited that the workers had imperfect knowledge, took insufficient preventive measures, and had weak susceptibility perceptions [33]. In another work, Shi et al. [34] investigated the present level of evidence-based chronic disease prevention (EBCDP) by taking interviews with health practitioners and patients of different health institutes in China and found that it was at an earlier level in the implementation of prevention practices. Further, a survey was conducted in Ukraine consisting of medical, custodial, and prison administrative staff with a sample size of 243 to determine criminal justice system workers’ attitudes towards drug addiction and opioid substitution therapy. The results demonstrated that the worker’s attitude was negative towards drug addiction [35].

Further, Mao and Yang [36] studied the expansion of infectious diseases among human beings and prevention practices to save themselves by making two networks. This infection network deals with disease transmission and a communication network that deals with preventive measures. Moreover, Przybyla et al. [37] conducted a study to assess the attitude, knowledge, and awareness of pharmacy students regarding human immunodeficiency virus (HIV) pre-exposure prophylaxis (PrEP). It was done by using descriptive statistics and multivariate logistic regression analysis. The results explored that educational modules’ progress helped increase exposure towards the attitude, information, and awareness regarding HIV and PrEP. Similarly, Ibrahim [38] investigated the expansion of HIV in Indonesia and focused on the prevention strategies to minimize it by renewing primary health care, paired with suitable economic and other risk units to health care. Given the survey of above-stated studies, the following hypothesis is formulated:

**Hypothesis** **1.** *Mythical attitude towards pandemic is likely to have a negative association with a willingness to adopt pandemic prevention.*

### 2.2. Pandemic Knowledge

Pandemic knowledge refers to awareness and the collection of information gained by individuals about a pandemic’s modes of transmission and prevention. It has been argued that different virus outbreaks like Ebola, Influenza, and Zika viruses could severely affect human beings, especially pregnant women. To this end, Krubiner et al. [39] explained twenty-two guidelines and recommendations that offer a road map for morally liable, socially unbiased, and deferential addition. This was done for the welfare of pregnant women and their offspring in the expansion and distribution of vaccination against pandemic outbreaks. Besides, a study was conducted in India between 2009 and 2015 to consider the impact of climate change on malarial pandemics and the influence of a specific area’s population, frequency, and prevalence of malarial parasite. Further, the seasonal variations were studied by using the logistic regression model. The results showed that the climate and seasonal change influenced pandemics as summers accelerated the pandemics, while winters had a significant negative effect [40]. According to Yang et al. [41], after SARS-2003 and MERS-2012, COVID-19 appeared as a new pandemic. Its main symptoms included dry cough, flu, temperature, and body pain. The Chinese government was reportedly taking measures for prevention and control as the human-to-human transmission rate was higher than SARS and MERS. It was suggested that there was a need to develop antivirals or vaccines that would offer a big opportunity. It was further opined that the virus was affecting the nation’s economy drastically. In light of these works, the following association is hypothesized:

**Hypothesis** **2.** *Pandemic knowledge is likely to have a positive association with a willingness to adopt pandemic prevention.*

### 2.3. Ease of Pandemic Prevention Adoption

Ease of pandemic prevention adoption refers to the availability of protective gears to individuals and the feasibility of practicing prevention measures such as lockdown and social distancing. A study was carried out to examine the feasibility of momentary ecological assessment by taking almost 21 respondents’ data. The results showed that momentary ecological assessment was easier and had no impact on behavior [42]. It has been estimated that almost 36.9 million people were affected by HIV/AIDS. Regardless of the facility of available drugs for disease treatment, lifetime therapy was required for its prevention and control and to avoid its re-emergence. Using biomedical tools, prophylaxis, and circumcision, the diffusion of HIV/AIDS could be controlled by the end of 2030 [43]. In another research, Spire et al. [44] discovered three essentials in the exertion to decrease the sexual diffusion of HIV/AIDS struggle deterrence lethargy, expand HIV checking and hostility, humiliation, and prejudice. It also contended for an indulgent damage lessening method to the deterrence of sexual diffusion of HIV that considered the clarification of danger by various persons and societies in the period of antiretroviral treatment. Lee et al. [45] analyzed the impact of information and communication technology usage on psychosocial factors by conducting a questionnaire survey from 394 U.S. residents. The feasibility of pandemic prevention was a significant contributor to future anxiety.

Moreover, Zhou et al. [46] conducted an online survey-based study in China’s Wuhan city, including 728 respondents, to analyze the influence factors of wearing face masks. The availability of face masks positively affected individuals’ behavior of wearing them. Intawong et al. [47] studied the role of application technology in Thailand in helping the COVID-19 patients and high-risk individuals to discover their disease symptoms through quick tracking strategies. In another work, Thomas et al. [48] assessed the role of technologies in facilitating the prevention of pandemics worldwide. To this end, social media, artificial intelligence, and other digital technologies helped to promote the ease of pandemic prevention. Clipper [49] also argued that tech solutions strengthened the healthcare systems and made prevention adoption easier through information communication. Further, Kusuma et al. [50] conducted a survey-based analysis in four South Asian countries (India, Pakistan, Bangladesh, and Sri Lanka) by recruiting 29,809 respondents to evaluate the feasibility of COVID-19 prevention adoption. The individuals were found less likely to adopt pandemic prevention due to the unavailability of protective gears. Finally, Irfan et al. [51] examined and revealed the negative impact of the unavailability of face masks on willingness to wear face masks in Pakistan. In view of the abovementioned literature, the following relationship is hypothesized:

**Hypothesis** **3.** *Ease of pandemic prevention adoption is likely to have a positive association with a willingness to adopt pandemic prevention.*

### 2.4. Self-Efficacy

Self-efficacy refers to individuals’ beliefs of handling or managing a certain situation. It describes individuals’ ability to carry out certain actions in the needful hours. Blue [52] explored the impact of attitude, beliefs of peer groups, and self-efficacy on diabetic patients’ intention to do physical activity and eat healthy food for prevention and control by taking a sample of 106 adults at risk of diabetes. The results explained that all the variables greatly influenced intentions to take a healthy diet and make oneself physically fit. Another work consisting of 147 nurses in Korea was conducted to explore the impact of attitude and self-efficacy on the nursing intention to look after patients in emerging transferrable syndromes using the theory of planned behavior. The findings indicated that the most effective variable to influence intentions was self-efficacy [53]. It has been argued that learning and forgetting behavior during pandemic disease was investigated by using the models such as the forgetting curve model (IFC), memory reception fading, and cumulating model (MRFC). It was done through sensitivity and simulation analyses. The results revealed that MRFC is more efficient and effective than IFC, which is suitable for fewer pandemics with a lower fatality rate [54]. Then, Aruta [55] analyzed and declared individuals’ resilience and financial issues as the strongest determinants of psychological distress in Filipino individuals. In another work, Chen et al. [56] examined and found an adverse influence of COVID-19 on medical staff’s mental health than Wuhan’s general public. Given the above-discussed studies, the hypothesized association is given as follows:

**Hypothesis** **4.** *Self-efficacy is likely to have a positive association with willingness to adopt pandemic prevention.*

### 2.5. Peer Groups’ Beliefs

Peer groups’ beliefs refer to the ways of thinking of an individual’s peers, including friends, colleagues, neighbors, and other people with whom the individual is often in contact. During a pandemic, their ways of thinking might influence the behavior of an individual. It has been narrated that it would be impossible to deal with a pandemic without public cooperation, irrespective of the number of physicians, technology, health care personnel, and medical facilities available. To bring public cooperation, governments, and high authorities’ participation was recommended because without considering the social dimension, it would not be possible to control the outbreak [57]. After the outbreak of SARS in 2002 to 2003, HIV/AIDS pandemics had a significant effect on the world over the subsequent decades. It exposed the substantial function of social norms, beliefs, and attitudes in determining people’s lifestyles in society. It drew attention towards taking preventive measures and controlling pandemic diseases [58]. Zhang et al. [59] examined and noted the negative influence of the COVID-19 pandemic on peer groups’ physical activities in the U.S. Moreover, a study consisted of Thai college undergraduate students employed via peer leaders to find how hypothetical variables function inside theory-based intermediation. It offered a concise HIV preventive measure plan to improve Thai college students’ knowledge regarding HIV/AIDS prevention and improve their confidence and motivation to fight against this disease [60]. In light of these studies, the following hypothesis is formulated:

**Hypothesis** **5.** *Peer groups’ beliefs is likely to have a positive association with willingness to adopt pandemic prevention.*

### 2.6. Moral Values

Moral values involve an individual’s sense of obligations and responsibility towards others. To illustrate, during the outbreak of a pandemic, taking care of others by helping them adopt prevention measures defines the moral values of individuals. Similarly, moral values also included an individual’s cooperation with others to facilitate them get through difficult times. Concerning society’s morality, a study was carried out to analyze the variations in tobacco usage and preventive measures taken by taking qualitative data from teachers of 12 schools of Maharashtra and Bihar [61]. The results discovered that tobacco usage was at a higher rate in Bihar as compared to Maharashtra as the moral norms strongly encouraged tobacco usage in Bihar. Besides, efficient functional resolutions to the difficulties between-group disagreements urged various ethical good fortune that fairly concerned Evo liberals, and not any of those social modernizations needed intervening at the stage of personal ethical capabilities. There were almost certainly believable worldwide settlements that might resolve the difficulties of anthropogenic atmospheric modification and worldwide scarcity [62]. In another research, Edmonson et al. [63] studied that eighty percent of nurses faced harassment in hospitals, and twenty-one percent of the turnover rate was also caused by bullying. There were many reasons involved, like difference in regions, gender, power, behavioral patterns, etc. The individuals experienced poor health and mental and physical stress in response to harassment. Prestia [64] examined the challenges faced by nurses during the international COVID-19 pandemic outbreak and found their pivotal role in keeping with the moral obligations to take care of patients. In the sense of contextual behaviors, Borges et al. [65] stated that the COVID-19 pandemic brought into light many moral dilemmas. Akram [66] reported that the U.S. healthcare system adopted utilitarian policies to deal with moral injuries during the COVID-19 pandemic outbreak. Liang et al. [67] studied and revealed respondents’ depressive behaviors and moral collapse from China’s Hubei province during the pandemic outbreak. Finally, Donnarumma and Pezzulo [68] figured out that moral collapse observed for the Italian citizens from a high outbreak region (Milan) to low outbreak regions (southern Italy) caused severe outbreak in those regions. It means moral decisions were significantly linked with the pandemic prevention measures’ adoption during the outbreak. Based on the abovementioned works, the following association is hypothesized:

**Hypothesis** **6.** *Moral values are likely to have a positive association with a willingness to adopt pandemic prevention.*

### 2.7. Risk-Averse Behavior

Risk-averse behavior is an individual’s tendency to avoid uncertain or risky situations. To illustrate, a risk-averse individual is reluctant to indulge in events with uncertain or risky outcomes. Thus, such individuals are more inclined towards prevention adoption during a pandemic. It has been shoen that some infections stay dormant in human beings without infecting them. However, some infectious diseases not only infect the human being in which they were living but also infect other human beings who come into contact with the carrier. In order to test the persons’ ability to evade the risk of the disease spreading, a pandemic spreading model was proposed by [69]. The findings showed that the cause of the expansion of disease was transforming dormant human beings into explosives. Also, self-prevention helped minimize the expansion of infectious diseases [69]. Further, Berry and Finnoff [70] investigated how individuals might react against the increasing pandemic by proposing two investment policies. Those policies included the adaptation policy (in which individuals can invest in domestic capital) and prevention policy (in which individuals can invest in foreign capital). In this way, the expansion of pandemics could be controlled. In the same vein, Lee and You [71] investigated and found a significant impact of health factors on the avoidance of healthcare use in South Korea. Hashiguchi et al. [72] analyzed the association among health risk, productivity, and work motivation among the construction workforce in Japan. The health risk was significantly associated with productivity and work motivation. Cordellieri et al. [73] studied the influence of psychological factors on COVID-19 containment and observed its negative impact. Moreover, there were three identified reasons that risk-averse behavior was considered as a distinct aim of health policy. First, public health security was a priority. Second, it was essential for societal planning. Finally, it was a suitable response towards decision-making, especially when available pieces of information were limited [74]. In light of these works, the following hypothesis is formulated:

**Hypothesis** **7.** *Risk-averse behavior is likely to have a positive association with a willingness to adopt pandemic prevention.*

### 2.8. Perceived Risk

Perceived risk demonstrates an individual’s subjective assessment of his/her risk of indulging in an adverse situation. In real life, perceived (subjective) risk plays a more substantial role than the actual (objective) risk in shaping the behavior of individuals [75]. Thus, the better the risk is perceived by an individual more likely he/she is to adopt pandemic prevention. It is the subjective opinion regarding the nature and magnitude of a risk encountered by the people. It is generally used for natural disasters and environmental or safety risks. Concerning this factor, Ho et al. [76] conducted a study in Taiwan in 2004 to discover the impact of perceived risk on the kind of tragedy like a flood or land sliding and characteristics of individuals (victims). The main results depicted that perceived risk has a significant influence on the type of disasters and characteristics of victims. A project named Highland Malaria Project was developed in Kenya and Uganda for early detection, control, and malaria prevention between 2001 to 2006. The main reason for this was to mitigate the risk of its expansion by detecting and curing it at an early stage [77]. From a different perspective of perceived risk, Valeeva et al. [78] studied the factors influencing the farmer’s risk management strategies named biosecurity and animal health programs as well as their perception in terms of the management of disease and animal health risks by taking data from 164 participants and using a structural equation modeling approach. The results indicated that biosecurity measures are more efficient as compared to animal health programs.

Moreover, Kiviniemi et al. [79] researched the influence of the education gap in the perceived risk of HIV by taking data from 1993 to 2000 in the U.S. The findings exposed that people with a low level of education are unaware of disease and health risk compared to people with a high level of education. Hence, the perceived risk is high for highly educated people as compared to less educated people. Similarly, Raude et al. [80] unveiled the perceptions relevant to risk and behaviors in the malarial pandemic outbreak results taking the data of 434 French Guiana residents. The results showed that the perceived risk of infection considerably reduces over time. After that, Rodriguez-Besteiro et al. [81] examined and revealed a significant influence of perceived pandemic risk on nutrition, psychology, and habits of Spanish individuals. Sica et al. [82] evaluated the influence of perceived COVID-19′s danger and anxiety on pandemic protection, and revealed its positive impact for 742 community members in the Italian context. In their research, Ding et al. [83] examined the COVID-risk perception in China and discovered that college students in Hubei province had a high level of risk perception. Finally, Li et al. [84] examined the impact of perceived risk on social support and the possibility of contracting COVID-19 by conducting an online questionnaire from 1970 Taiwan’s residents. It was found that perceived risk mediated the impact of social support on the possibility to contract the COVID-19 disease. These studies lead to the formulation of the following hypothesis:

**Hypothesis** **8.** *Pandemic knowledge is likely to have a positive association with willingness to adopt pandemic prevention.*

### 2.9. Lack of Trust in Political Will

A lack of trust in political will refers to the absence of individuals’ confidence in political institutions, which damages his/her belief in the righteousness of these institutions. If such confidence is lacking, individuals would be likely to demonstrate civil disobedience and be reluctant to follow pandemic prevention guidelines by the governments. It has been suggested that the government plays a major role in reducing obesity, communicable, non-communicable diseases, and increasing the health conditions of the public. For this purpose, the monitoring and evaluation system was advised to be introduced to test the policies made by the government sector. It was done to make a healthy food environment like a government healthy food environment index developed in collaboration with international experts to maintain a hygienic food environment and reduce obesity [85]. Moreover, Yu et al. [86] analyzed the impact of government-controlled payment on the government’s health services to the general public in Shanghai, China. The Shanghai government focused on developing community health services, which offered health services to the general public in 1997. Nevertheless, their main purpose was to make a profit instead of providing excellent services to the general public. In order to resolve the issue, the government introduced the government-controlled payment process that focused on providing excellent services instead of making a profit, and it positively influenced the provision of quality services to meet the health requirements of people. Moreover, health officers’ hand hygiene was an important factor in preventing and controlling disease transmission from patient to patient or healthy person. Allegranzi and Pittet [87] focused on promoting hand hygiene and issues faced by health workers in adopting alcohol-based hand wash to reduce healthcare-associated infections. In light of the above reviewed literature, the following hypothesis is developed:

**Hypothesis** **9.** *Lack of trust in political will is likely to have a negative association with willingness to adopt pandemic prevention.*

## 3. Materials and Methods

### 3.1. A Hybrid Theoretical Framework

This work extends the planned behavior (PBST) and the reasoned action school of thoughts (RAST) by incorporating new intention-based critical factors (ICFs). The new framework is called the hybrid theoretical framework. RAST was postulated by Fishbein and Ajzen [88]. They advanced the notion that the actions of individuals complied with their intentions. People anticipate the perception-based influence of their activities instead of immediately executing real actions. Hence, people tend to perform actions that they feel will contribute to positive outcomes. In this fashion, two dimensions are involved in determining the behavior based on individuals’ willingness to adopt pandemic prevention: (i) mythical attitude towards pandemic and (ii) peer groups’ beliefs. The attitude is defined as individuals’ common sense-based confirmation or disconfirmation of behavioral intention [89]. The composition of individuals’ attitudes towards pandemic prevention may stem from a set of values they have, and the appraisal of consequences associated with the behavioral intention. In addition, peer groups’ beliefs can be explained as a collection of expectations of how others evaluate a person’s actions and motivations [90].

**Figure 1:**
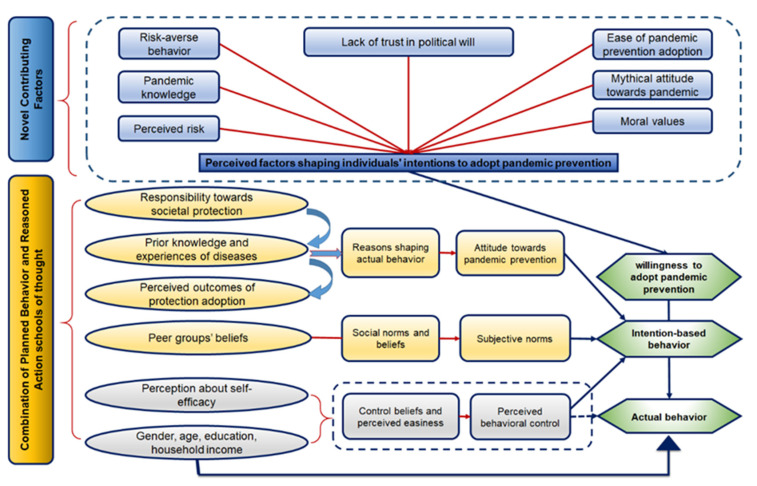
Modifications to the planned behavior and reasoned action schools of thought for novel contributing factors affecting individuals’ willingness to adopt pandemic prevention. Source: Authors’ drawing.

Originally, RAST was thought to be entirely composed of intention-based behaviors formed by the attitude towards some action and peer groups’ beliefs. Afterward, an influential opinion came forth, referring that intention was not independently developing individuals’ behavior, but some control factors were also involved. In this regard, Ajzen [90] presented a modified RAST version by including a novel self-efficacy element and characterized it as PBST (Figure 1). Self-efficacy is described as the power that people feel to have for executing some action. Besides, control beliefs and feasibility are the fundamentals of self-efficacy. The control beliefs are based on individuals’ intention to have or lack the ability and knowledge to do something. In parallel, feasibility involves people’s judgment about the convenience of executing some action [90].

RAST and PBST are commonly used to identify multifaceted intention-based behaviors in behavioral studies [91,92]. This research advances the RAST and PBST behavioral paradigms to augment them for some novel ICFs. Among those factors, peer groups’ beliefs, pandemic knowledge, self-efficacy, and attitude were used in mainstream works [91,93]. However, factors like perceived risk, risk-averse behavior, moral values, ease of pandemic prevention adoption, and lack of trust in the political will are not known to be incorporated in a behavioral framework, a combination of RAST and PBST. Thus, the present research developed this new framework incorporated those factors to demonstrate their linkages with individuals’ WAPP (Figure 1). The content analysis of empirical literature was done to detail the foundation of those factors provided in the Supplementary Materials.

Using a hybrid theoretical framework, this work investigates Pakistanis’ local intention-based WAPP translating it to the global context during the COVID-19 outbreak. In this regard, as per previous studies [93,94], behavioral intention has been considered instead of actually experienced behavior. Finally, the social and demographic features such as gender, age, education, and household income are taken as the controls, which partially contribute to the perceived behavioral control.

### 3.2. Survey-Based Data Compilation

A questionnaire was designed and shared with the health counselors and advisors (from the National Institute of Health), medical practitioners (from Shifa International Hospital, Pakistan Institute of Medical Sciences, and Aga Khan University Hospital), professors, and associate professors (from Quaid-i-Azam University, King Edward College, and Forman Christian College University) from the fields of Sociology, Medicine, and Psychology to obtain their expert feedback for pre-examination purposes. These expert participants played a dual role in the assessment of the questionnaire. Firstly, they commented on the contents of the questions to improve their clarity and quality. It established the content validity of the questionnaire. Secondly, they filled in the questionnaire for pilot testing to verify the functionality of the questionnaire. It established the face validity of the questionnaire [95]. The profiles of the participatory role-playing individuals are given in Appendix A (Table A1).

A questionnaire in English was combined with Urdu translation format removing any language barriers and producing informed feedback. This online survey was conducted in Pakistan during May–June 2020. In the face of the ongoing pandemic outbreak, the questionnaire was floated in numerous Facebook (Facebook Inc., Menlo Park, CA, USA) and WhatsApp (WhatsApp Inc., Menlo Park, CA, USA) groups among the social circles of friends, friends’ friends, colleagues, colleagues’ friends, and scholars and students from universities across universities. Ethical considerations were included by stating the research aims and scope in the questionnaire’s introductory paragraph to ensure the respondents’ informed consent. Furthermore, the confidentiality and anonymity of respondents were also guaranteed during the questionnaire conduction. Following Kamenidou et al. [96], the questionnaire conduction process was based on mixed non-probability sampling, which involved convenience, snowball, and criteria sampling procedures. The recruitment criterion was mainly based on the age of the respondents. Respondents below 18 years of age were advised not to fill in the questionnaire. Also, the individuals reluctant to provide their consent were excluded. (i.e., exclusion criteria). Moreover, the respondents needed to be residents of Pakistan. Further, since the questionnaire was conducted online, respondents on social media (Facebook and WhatsApp) were the only population available to generate the data sample (i.e., inclusion criteria). The respondents were from heterogeneous backgrounds in terms of occupation, qualification, and household income, among other traits. It considerably led the findings to be generalized for the population belonging to heterogeneous backgrounds. The survey was conducted from a total of 931 respondents. After initial scrutiny, 828 questionnaires were found completely and appropriately filled in by the respondents. Those questionnaires were declared valid for analysis purposes. The designed questions are presented in Appendix B (Table A2).

### 3.3. Data and Statistical Analysis

**Figure 2:**
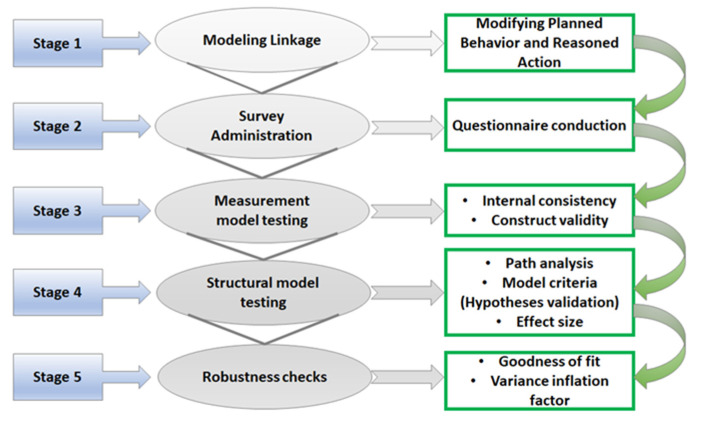
Schematic outline of the research methodology. Source: Authors’ elaboration.

The partial least squares (PLS)-based path model is adopted to assess the ICFs impacting individuals’ WAPP. A Likert scale consisting of five-points included 5 = “Totally agree”, 4 = “Agree”, 3 = “Neutral”, 2 = “Disagree”, and 1 = “Totally disagree.” The schematic outline of the research methodology is presented in Figure 2.

#### 3.3.1. Demographic Data

Data on the demographic characteristics of the respondents are reported in Table 1. The participation of males (66.5%) was higher than that of females (33.4%). The proportion of urban respondents (59.3%) exceeded that of rural respondents (40.7%). The main proportion of respondents (54.7%) consisted of youth (up to 25 years old), while middle-aged individuals (26–50 years) made the second-largest age group (31.3%). The mean of respondents’ age was 30.26 years, while its standard deviation was noted as 12.86. The respondents varied from illiterate (zero schooling years) to postgraduate (18 and above schooling years) in qualification. Bachelors (14 schooling years) made the largest proportion (20.9%), followed by the secondary (10 schooling years) and the higher secondary (12 schooling years) groups. The smallest proportion (4.2%) was based on illiterate respondents (zero schooling years). The largest proportion of respondents (56.6%) was unmarried, while a tiny proportion (2%) was divorced. The majority of respondents (34.2%) were employees in both public and private sectors, while students comprised the next significant share (31.3%). However, labor contributed to the smallest proportion (14.6%). The highest percentage of the respondents (43.4%) were from households with upper middle income (300,001–600,000 PRK per annum), while the lowest income households were in the smallest proportion (5.4%).

#### 3.3.2. Statistical Measurement Model

Confirmatory factor analysis was carried out to explore whether the models were reliable and valid. The assessment of external loadings was conducted and is shown in Table 2. The external loading equivalent to or greater than 0.7 was argued to determine variations roughly surpassing 50% [97], showing that the calculated factor attained a permissible degree of reliability. As a result, external loading values above 0.7 suggest the non-exclusion of the loading factor [98].

Moreover, [99] suggested that non-external consistencies depict the reliability of a construct. In this respect, ρ-A, Cronbach-alpha (C-α), and composite reliability (CR) were employed. The range of values from 0.7 through 0.95 suggests satisfactory reliability [100]. Since C-α may understate a finite sample’s efficiency, the use of an additional CR measuring tool is encouraged [101]. Furthermore, the magnitudes of ρ-A in a range between CR and Cronbach-alpha are taken to be accurate [102]. The average variance extracted (AVE) is reported in Table 2. Hair et al. [103] suggested that AVE surpassing 0.5 can be considered reliable, which is true in the present case. Thereby, the constructs in Table 2 are reliable. These findings authenticated the convergent validity and reliability of our measurement model.

As a step further, the confirmation of discriminant validity is crucial for assessing the scientific data’s authenticity. Ketchen [104] suggested that the discriminant validity required the cross-correlations between latent constructs (LTCs) to be less than their reflective (self) correlations. In the present case, cross-correlation values of all constructs were less than their reflective correlation values (Table 3). Following Hair et al. [105], the discriminant validity is satisfied based on this criterion. Moreover, an advanced discriminant validity test by Henseler et al. [102] is used for further verification. This is known as the heterotrait-monotrait ratio (HMR) of correlations. It calculated the pairwise cross-correlations between the constructs (Table 4). At 90% confidence interval, all the cross-correlations are found within the range of confidence interval, demonstrating that the discriminant validity is established. HMR is the most recent test and it has been reported to perform better than the Fornell-Larcker [102] criterion. Since the discriminant validity is proved valid, the path analysis can be carried out.

## 4. Main Results

**Figure 3:**
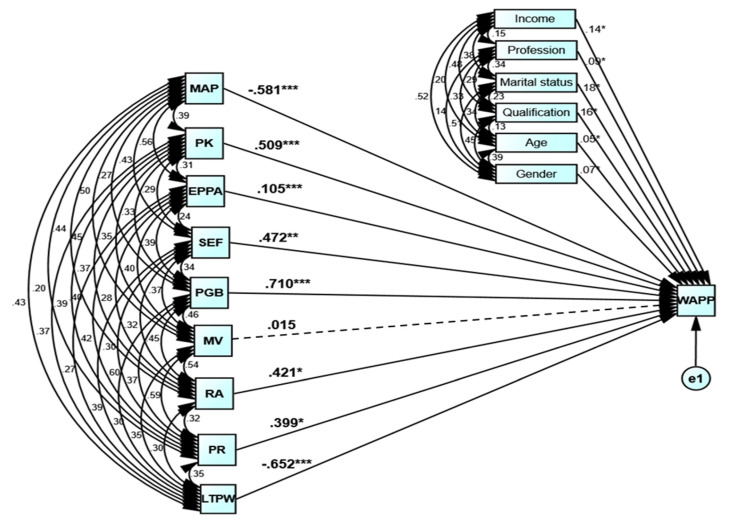
Path modeling-based estimation of coefficients. Notes: * *p* < 0.10, ** *p* < 0.05, *** *p* < 0.01. Solid line denotes significant path, while dashed line denotes insignificant one. Source: Authors’ elaboration.

The path modeling-based results are shown in Table 5 and Figure 3. The structural model was evaluated after the measurement model were proven to be reliable and efficient. As a primary condition, the R-square was generated for each of the constructs. R-square measures the variations captured by each of the non-exogenously discovered constructs to communicate the structural model’s predictive capacity. As a rule of thumb, a magnitude no less than 0.25 has been proposed to be an average score, whereas a magnitude below 0.13 is insufficient to pass this criterion in the behavioral sciences. In contrast, the badness of outcome is exhibited by any score less than or equal to 0.03 [48]. In the present case, the R-square value is 0.807, which is well above 0.25, satisfying the path model’s first criterion (Table 5).

Next, Stone–Geisser’s Q-square criterion was used explore the LTCs’ predictive relevance [107,108]. The non-negative range score reflects the LTCs’ predictive relevance [102]. Further, the predictive relevance’s relative impact is given by the degree of this criterion. A Q-square > 0.35 indicates that the exogenous constructs imparted adequate prediction for their respective endogenous constructs [97]. The magnitude of the measured Q-square (0.365) proved the accuracy and precision of the structural model. The path coefficients analysis is taken as a further prerequisite. In the structural model, an approximate path coefficient score above 0.1 indicates a significant contribution of a respective variable to the dependent variable [103]. After that, f-square is obtained, determining the effect size to characterize a construct’s contributing ability. Based on the f-square score, exogenous constructs define the identified differences in endogenously defined LTCs [109].

The path modeling does not require the prior existence of a normal distribution principle. Alternatively, this principle is followed by the bootstrap-based parameter estimation method to evaluate the importance of external loading and ICFs’ path coefficients. The bootstrapping method scrutinizes nearly 4 × 10^4^ samples extracted from the initial sample using the “with replacement” alternative for estimating every bootstrapped sample. This bootstrapping procedure involves generating a probability distribution for manipulating the variances and standardized residuals. To assess the validity of path coefficients, the null hypothesis of $H_{1}$ = $H_{2}\;$ = $H_{3}$ = $H_{4}$ = $H_{5}$ = $H_{6}$ = $H_{7}$ = $H_{8}$ = $H_{9}$ = 0 was assessed against the alternative of $H_{1}$ ≠ $H_{2}\;$ ≠ $H_{3}$ ≠ $H_{4}$ ≠ $H_{5}$ ≠ $H_{6}$ ≠ $H_{7}$ ≠ $H_{8}$ ≠ $H_{9}$ ≠ 0. For decision-making, the probabilities equal to or less than the statistical magnitude of 0.05 are considered significant at a 5 percent level [102]. To this end, the only null hypothesis retained was $H_{6}$ = 0, while the remaining were successfully rejected (Table 5). In other words, all the ICFs contributed to the WAPP of individuals, except for the moral values.

**Figure 4:**
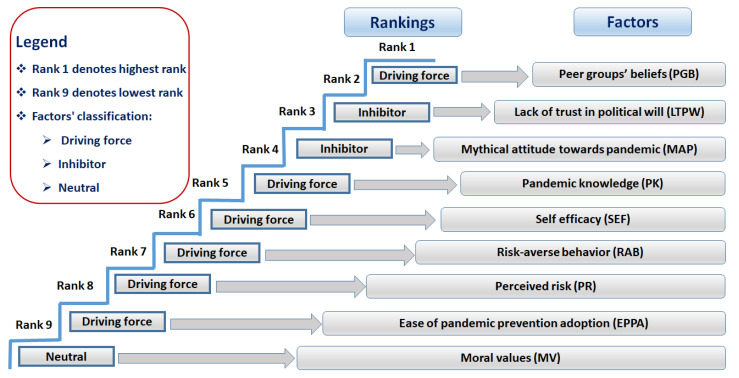
Ranking the significance of intention-based critical factors (ICFs) affecting individuals’ willingness to adopt pandemic prevention (WAPP). Source: Authors’ elaboration.

The path coefficients-based relative significance of the ICFs of individuals’ WAPP is depicted in Figure 4. The ICF of peer groups’ beliefs is highest ranked, followed by a lack of trust in political will, mythical attitude towards pandemic, and so on. The moral values are the lowest-ranked ICF. This ranking of significance is based on the strength of the path coefficients. For illustration, the magnitudes of path coefficients are provided as peer groups’ beliefs = 0.710, lack of trust in political will = 0.652, mythical attitude towards pandemic = 0.581, pandemic knowledge = 0.509, self-efficacy = 0.472, risk-averse behavior = 0.421, perceived risk = 0.399, and ease of pandemic prevention adoption = 0.105. However, the coefficient of moral values remained insignificant and lowest (0.015). And thus, moral values imparted a neutral contribution to the individuals’ WAPP.

**Figure 5:**
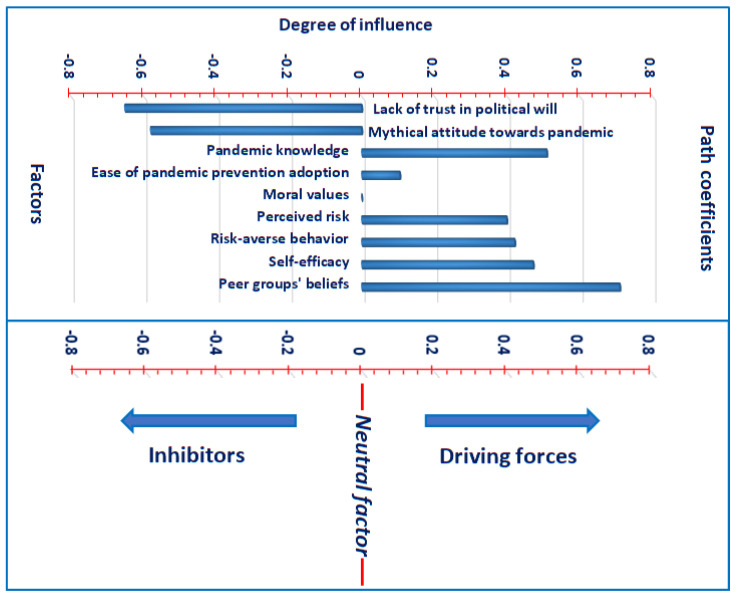
Path coefficients-based classification of factors into driving forces, inhibitors, and neutral factors. Source: Authors’ elaboration.

In summary, a lack of trust in the political will and a mythical attitude towards the pandemic are the dominant inhibitors of individuals’ WAPP. Meanwhile, the other ICFs are revealed as the driving forces of individuals’ WAPP, except moral values which highlighted a neutral role in determining the individuals’ WAPP. Peer groups’ beliefs and pandemic knowledge are discovered as the main driving forces of individuals’ WAPP (Figure 5).

## 5. Discussion, Limitations, and Future Research Directions

### 5.1. Discussion

In the present research, pandemic knowledge played a positive role in escalating the individuals’ WAPP. It means that if individuals are aware of the fatal and lethal aspects of a pandemic, they are willing to protect themselves from it. In contrast, a survey-based study of 740 patients in Jordan investigated and revealed that most participants had knowledge and awareness about Chronic Kidney Disease, but half of them had the wrong information and could not detect its symptoms at the initial level. Thus, their knowledge affected the adoption of prevention practices negatively [110]. However, analogous to our results, a study on 265 Black faith leaders in the U.S. found that increased awareness regarding HIV through print and social media, church websites, and making policies of HIV prevention could help reduce the disease [111]. It was further argued that the treatment approach and treatment knowledge were essential role player in preventing the spread of HIV around the world [112]. Along these lines, the dissemination and acquisition of correct and well-informed pandemic knowledge could play an integral driving influence during pandemic outbreaks.

The Ebola virus spread through African countries in 2014, giving rise to increased fatality rates. The main reason behind the pandemic’s spread was the increased population mobility worldwide (domestic and international), lack of awareness, and weak health systems. The lesson learned from the last pandemic was that a country should make its health system better. Vaccination-based treatment, safety policies, advertisement on pandemic prevention, and pandemic prevention impacts were emphasized [113]. The mythical attitude towards the pandemic proved to be a bottleneck in enhancing the individuals’ WAPP. This finding was consistent with that of Khalil and Abdalrahim [110], who revealed a negative influence of attitudinal construct on disease prevention practices. Similar to the findings of the present work, Liao and Wang [114] evaluated and uncovered a supportive influence of epidemic information on China’s prevention adoption. In the same vein, Ritter et al. [93] explored the ways through which farmers adopted the policies based on management practices for the prevention and control of diseases. Social relationships, social media, and farm consultants’ recommendations also motivate the farmers to adopt such practices for prevention and control.

Our results revealed that peer groups’ beliefs and self-efficacy positively drove the individuals’ WAPP. Similarly, a different study conducted in four regions, including Toronto, Guangdong, Singapore, and Hong Kong, evaluated the beliefs of peer groups and self-efficacy on preventive behaviors to prevent and control the SARS pandemic in these regions. However, the results demonstrated that self-efficacy was not a substantial predictor for all respondents in Guangdong [115]. Additionally, successions of the cholera pandemic outbreak in Hanoi interjected a flash of financial and economic triumphalism in the past changeover. In search of the basis of a rebellious syndrome linked with scarceness and less growth and expansion, media, official groups, and residents not only found victims but also offered a way out. They also permitted specific revelations of moral conduct, the public’s health, and societal order. In this regard, the beliefs of peer groups and self-efficacy strengthened the pandemic prevention adoption during the outbreaks [116].

This work has demonstrated the driving influence of perceived risk and risk-averse behavior in promoting individuals’ WAPP. Along these lines, Botzen et al. [117] discovered the impact of influence factors to prevent flood damage in New York. For this purpose, the protection motivation theory was taken as a theoretical base. Their results unveiled that factors such as attitude towards risk and time preferences played a major role in individuals’ decision-making regarding preventing and controlling floods in high-risk areas. It has been documented that health policy was necessary for the prevention and control of pandemics. Risk-averse behavior was considered a useful means to avoid pandemics. Further, Omodior et al. [118] investigated the impact of perceived severity and perceived susceptibility on the adoption of personnel protective behaviors (PPB) in the case of five mosquito-borne pandemics. They did it by considering a sample of 1043 respondents from the U.S. The diseases included West Nile virus, Dengue fever, Zika virus, Chikungunya, and Malaria. The outcomes disclosed that perceived severity was found among all mosquito-borne pandemics. Also, the people were more concerned about the adoption of PPB in the cases of Zika virus, Chikungunya, and Dengue fever than in the cases of West Nile virus and Malaria. Finally, Cui et al. [119] conducted a survey to bridge a gap between the linkage between risk perception about avian-influenza and adoptive biosecurity measures (ABM) by taking a sample of 426 poultry farmers in China. The results discovered that increased perceived risk induced more ABM adoption. This finding is aligned with our results since perceived risk proved to be the driving force of individuals’ WAPP.

We found that ease of pandemic prevention adoption promoted the individuals’ WAPP. Consistent with our results, Kusuma et al. [50] revealed that the unavailability of protective gears (mainly hand sanitizers and face masks) adversely impacted the COVID-19 prevention adoption in four South Asian countries (India, Bangladesh, Sri Lanka, and Pakistan). It means that the easier the adoption of pandemic prevention, the more that individuals will be willing to adopt it. Furthermore, Yang et al. [120] conducted an impact analysis between the feasibility of adopting good agricultural practices by the small farmers and enhancing raw milk hygiene by taking data from 34 farms. The results indicated that almost 47.73% of farmers were adopting hygienic policies for raw milk in the face of their feasible adoption.

We also revealed that a lack of trust in political inhibited the individuals’ WAPP. In support of this finding, past research found that E-guidelines and price premium by the government were some factors that positively influenced the adoption of hygienic practices by building the trust of farmers in political institutions [120]. Similarly, Cui et al. [121] studied the critical factors influencing Chinese poultry farms in response to the avian influenza virus by taking semi-structured interviews from twenty-five poultry farmers between November 2016 and May 2017 using grounded theory. The results showed that the government must inform farmers regarding prevention and control at an early stage of the avian influenza virus through the proper communication networks. In contrast to our results, Paolini et al. [122] studied and discovered a positive contribution of political trust to COVID-19 distress in the Italian context. Similarly, Sarkar et al. [123] conducted a situation analysis in eight South Asian countries and confirmed that governance maximization was the optimal tool for preventing and controlling the COVID-19 epidemic.

### 5.2. Limitations and Future Research Directions

Since there is always room for improvement, this work has some limitations that can be overcome by future works. First, this study’s sampling procedure was not purely randomized which would limit its findings’ generalizability. It was not possible to make it strictly random due to the ongoing pandemic outbreak across the country. Therefore, some selected platforms were chosen to collect data through questionnaires. Future studies should overcome this limitation to make the sampling generation process purely random to gain enough generalizability of the findings. Second, this work has considered the case of intention-based factors during the ongoing pandemic outbreak; however, it cannot provide a complete picture of individuals’ behavior before and after the pandemic. Therefore, future studies should conduct a pre-and post-pandemic analysis to get a clear idea of how it affects the intention-based factors influencing the individuals’ adoption behavior. Third, this work analyzed the whole dataset, including rural and urban respondents. Future studies should also analyze the urban and rural samples separately to investigate the differences in individuals’ intention-based factors across the two samples. This would enhance the insight of the findings, providing a deep understanding of rural-urban heterogeneity. Fourth, there might exist possible dependencies among the constructs of this study. However, we have not considered this aspect since it needs to establish a separate model to incorporate the mediation or moderation impacts. Therefore, future works should include this aspect to analyze the potential mediation or moderation among those constructs. As a final point, this work merely conducted aggregated analysis without distinguishing the demographic features of the study sample. Future studies may consider disaggregated analysis for people under different age cohorts, different income groups, and across varying levels of qualification to see the differences of response across groups of individuals with heterogeneous demographic attributes. It would provide a rich and comparative analysis for more informed and targeted public health policy outcomes.

This work’s outcomes are unique in terms of reflecting the individuals’ intention-based driving forces, inhibitors, and neutral factors of WAPP from the perspective of a hybrid theoretical framework based on the planned behavior and reasoned action schools of thought. The consideration of ICFs is vital in the face of the fact that these factors significantly influence the intention of individuals to adopt preventive measures during pandemic spread, such as the currently ongoing outbreak of pandemic COVID-19. During the outbreak of an infectious pandemic, everyone’s participation to avoid viral transmission is critical. This work’s implications are useful guidelines on ICFs to shape the WAPP of individuals in Pakistan and at the global level during the outbreak of COVID-19 and potential future pandemics.

## 6. Conclusions

The key conclusion points are as follows: The peer groups’ beliefs, self-efficacy, risk-averse behavior, pandemic knowledge, ease of pandemic prevention adoption, and perceived risk were revealed to be the driving forces of the individuals’ willingness to adopt pandemic prevention. The inhibitors included the lack of trust in political will and a mythical attitude towards pandemic. However, moral values had a neutral role. Regarding the relative significance of intention-based critical factors, peer groups’ beliefs, as well as the lack of trust in the political will, were ranked the highest. Simultaneously, the moral values factor was ranked the lowest in determining individuals’ willingness to adopt pandemic prevention.

Based on the empirical results, the following policies are suggested. (1) The government should play a critical role at the central level (federal/provincial level) and the decentralized levels, including divisional, district (sub-division), Tehsil (district’s sub-division), and union council (Tehsil’s sub-division) levels, to win the trust of people at the grass-roots level. To this end, the government needs to develop and successfully implement favorable policies to improve its image in the public’s eyes. If people realize that the government is performing well, they will listen to the government’s guidelines in case of potential future pandemics. (2) The mythical attitudes of individuals lead them to refuse the adoption of pandemic prevention. Therefore, awareness campaigns on lethality and fatality of the pandemic must be organized, addressing this concern at all societal levels. Testing of communicable diseases such as COVID-19 at the grass-roots level may help remove individuals’ mythical attitudes regarding the disease’s existence. The mythical attitude is nurtured in the roots of culture. To uproot and modify such attitudes, education is the optimal solution, reshaping the behaviors of individuals in times of pandemics like COVID-19. Pandemic knowledge is something that promotes the adoption behaviors; therefore, individuals must be educated about the existence and transmission mechanisms of this pandemic irrespective of their age groups and income classes. Moreover, the government should expand the health sector’s capacity, and job creation should be enhanced. More employed individuals in this sector will help educate the people about such fatal epidemics’ seriousness.

(3) Perceived risk and risk-averse behavior were found be to among the significant contributors to individuals’ willingness to adopt pandemic prevention. It means that once individuals recognize the pandemic’s seriousness, vulnerability, and fatality, their objective of adopting pandemic prevention is strengthened. The high level of risk perception of communicable diseases (such as COVID-19) will substantially reform the individual’s willingness to adopt pandemic prevention. (4) The ease of pandemic prevention adoption was proved a significant driving force in determining the willingness of individuals to adopt the prevention. It implies that the easier the adoption of pandemic prevention, the higher the individuals’ willingness to adopt such preventative measures. Pandemic prevention gear like surgical masks, hand sanitizers, and hand wash soaps are not affordable for every individual in society. Therefore, to promote individuals’ WAPP, the provision of such protective measures free of cost or at discounted rates would aid in the adoption of pandemic prevention.

## Figures and Tables

**Table 1:** Attributive profiles of the respondents.

Attributes	Number	Contribution (%)
Gender		
Male	551	66.5
Female	277	33.4
Resident type		
Rural (village)	337	40.7
Urban (city)	491	59.3
Age		
Youth (up to 25 years)	453	54.7
Middle aged (26–50 years)	259	31.3
Old-age (more than 50 years)	116	14.0
Qualification (schooling years)		
Illiterate (0 years)	35	4.2
Primary (5 years)	69	8.3
Middle (8 years)	112	13.5
Secondary (10 years)	151	18.2
Higher secondary (12 years)	128	15.5
Bachelor (14 years)	173	20.9
Master (16 years)	119	14.4
Postgraduate (18 years and above)	41	4.9
Marital status		
Married	342	41.3
Unmarried	469	56.6
Divorced	17	2
Profession		
Self-employed	165	19.9
Labor	121	14.6
Employees	283	34.2
Students	259	31.3
Household income (annual)		
Low (Up to 50,000 PKR)	143	17.3
Lower middle (50,001–150,000 PKR)	116	14.0
Middle (150,001–300,000 PKR)	218	26.3
Upper middle (300,001–600,000 PKR)	306	36.9
High (More than 600,000 PKR)	45	5.4

**Table 2:** Measurement model results.

Latent Constructs	Observed Variables	External Loadings	C-α	ρ-A	CR	AVE
MAP	MAP_1_	0.792	0.762	0.785	0.818	0.770
	MAP_2_	0.765				
	MAP_3_	0.819				
	MAP_4_	0.833				
	MAP_5_	0.781				
PK	PK_1_	0.802	0.786	0.803	0.867	0.794
	PK_2_	0.775				
	PK_3_	0.793				
	PK_4_	0.812				
	PK_5_	0.726				
	PK_6_	0.799				
	PK_7_	0.845				
	PK_8_	0.756				
EPPA	EPPA_1_	0.751	0.725	0.792	0.811	0.746
	EPPA_2_	0.773				
	EPPA_3_	0.795				
	EPPA_4_	0.728				
SEF	SEF_1_	0.788	0.784	0.819	0.886	0.798
	SEF_2_	0.823				
	SEF_3_	0.795				
	SEF_4_	0.776				
	SEF_5_	0.861				
PGB	PGB_1_	0.735	0.793	0.826	0.844	0.819
	PGB_2_	0.789				
	PGB_3_	0.802				
	PGB_4_	0.826				
MV	MV_1_	0.794	0.765	0.789	0.823	0.771
	MV_2_	0.774				
	MV_3_	0.832				
	MV_4_	0.769				
	MV_5_	0.734				
RAB	RAB_1_	0.797	0.824	0.841	0.873	0.835
	RAB_2_	0.824				
	RAB_3_	0.800				
	RAB_4_	0.775				
	RAB_5_	0.730				
PR	PR_1_	0.818	0.805	0.839	0.857	0.827
	PR_2_	0.836				
	PR_3_	0.794				
	PR_4_	0.722				
	PR_5_	0.765				
	LTPW_1_	0.877	0.792	0.813	0.833	0.804
	LTPW_2_	0.810				
LTPW	LTPW_3_	0.848				
	LTPW_4_	0.725				
	LTPW_5_	0.769				
WAPP	WAPP_1_	0.744	0.821	0.849	0.886	0.834
	WAPP_2_	0.829				
	WAPP_3_	0.790				
	WAPP_4_	0.764				
	WAPP_5_	0.893				
	WAPP_6_	0.745				

Notes: Degree to agree with the affirmative response is classified as: 5 = “Totally agree”, 4 = “Agree”, 3 = “Neutral”, 2 = “Disagree”, 1 = “Totally disagree.” C-α: Cronbach-alpha. MAP: Mythical attitude towards pandemic, PK: Pandemic knowledge, EPPA: Ease of pandemic prevention adoption, SEF: Self-efficacy, PGB: Peer groups’ beliefs, MV: Moral values, RAB: Risk-averse behavior, PR: Perceived risk, LTPW: Lack of trust in political will, WAPP: Willingness to adopt pandemic prevention. AVE: average variance extracted, CR: composite reliability, ρ-A: internal consistency reliability, C-α: Cronbach-alpha.

**Table 3:** Discriminant validity results based on Fornell and Larcker [106] criterion.

Factors	MAP	PK	EPPA	SEF	PGB	MV	RAB	PR	LTPW	WAPP
MAP	(0.88)									
PK	0.198	(0.75)								
EPPA	0.203	0.327	(0.76)							
SEF	0.511	0.295	0.197	(0.85)						
PGB	0.136	0.189	0.205	0.329	(0.79)					
MV	0.376	0.143	0.428	0.312	0.298	(0.83)				
RA	0.281	0.451	0.365	0.408	0.156	0.396	(0.89)			
PR	0.372	0.268	0.272	0.216	0.381	0.401	0.415	(0.86)		
LTPW	0.490	0.311	0.290	0.345	0.410	0.348	0.264	0.255	(0.89)	
WAPP	0.277	0.506	0.317	0.437	0.178	0.273	0.367	0.316	0.307	(0.82)

**Table 4:** Discriminant validity testing based on the Heterotrait-Monotrait Ratio.

Factors	MAP	PK	EPPA	SEF	PGB	MV	RAB	PR	LTPW
MAP									
PK	0.70 CI_0.90_ [0.68;0.72]								
EPPA	0.64 CI_0.90_ [0.62;0.67]	0.69 CI_0.90_ [0.67;0.71]							
SEF	0.65 CI_0.90_ [0.63;0.68]	0.63 CI_0.90_ [0.61;0.65]	0.74 CI_0.90_ [0.71;0.76]						
PGB	0.76 CI_0.90_ [0.73;0.78]	0.71 CI_0.90_ [0.69;0.73]	0.73 CI_0.90_ [0.71;0.75]	0.75 CI_0.90_ [0.73;0.77]					
MV	0.68 CI_0.90_ [0.66;0.70]	0.66 CI_0.90_ [0.64;0.68]	0.71 CI_0.90_ [0.69;0.73]	0.74 CI_0.90_ [0.72;0.76]	0.69 CI_0.90_ [0.67;0.71]				
RA	0.73 CI_0.90_ [0.71;0.75]	0.76 CI_0.90_ [0.74;0.78]	0.65 CI_0.90_ [0.63;0.67]	0.62 CI_0.90_ [0.60;0.64]	0.67 CI_0.90_ [0.65;0.69]	0.69 CI_0.90_ [0.67;0.71]			
PR	0.64 CI_0.90_ [0.62;0.66]	0.67 CI_0.90_ [0.65;0.69]	0.74 CI_0.90_ [0.72;0.76]	0.71 CI_0.90_ [0.69;0.73]	0.75 CI_0.90_ [0.73;0.77]	0.69 CI_0.90_ [0.67;0.71]	0.78 CI_0.90_ [0.76;0.80]		
LTPW	0.81 CI_0.90_ [0.79;0.83]	0.78 CI_0.90_ [0.76;0.80]	0.75 CI_0.90_ [0.73;0.77]	0.77 CI_0.90_ [0.75;0.79]	0.73 CI_0.90_ [0.71;0.75]	0.75 CI_0.90_ [0.73;0.77]	0.71 CI_0.90_ [0.69;0.73]	0.84 CI_0.90_ [0.82;0.86]	
WAPP	0.85 CI_0.90_ [0.83;0.87]	0.88 CI_0.90_ [0.86;0.90]	0.84 CI_0.90_ [0.82;0.86]	0.83 CI_0.90_ [0.81;0.85]	0.87 CI_0.90_ [0.85;0.89]	0.86 CI_0.90_ [0.84;0.88]	0.79 CI_0.90_ [0.77;0.81]	0.74 CI_0.90_ [0.72;0.76]	0.69 CI_0.90_ [0.67;0.71]

Notes: CI: confidence interval. The brackets [] contain the confidence intervals at 90%.

**Table 5:** Path modeling analysis and post-estimation criteria results.

Hypothesis	Hypothesized Path			PC	Assessment	VIF	f-Square	R-Square	Q-Square
H1	MAP	→	WAPP	−0.581 ***	Verified	2.429	0.405	0.807	0.365
H2	PK	→	WAPP	0.509 ***	Verified	4.274	0.355		
H3	EPPA	→	WAPP	0.105 ***	Verified	1.992	0.073		
H4	SEF	→	WAPP	0.472 **	Verified	2.651	0.329		
H5	PGB	→	WAPP	0.710 ***	Verified	2.843	0.495		
H6	MV	→	WAPP	0.015	Not verified	3.701	0.010		
H7	RAB	→	WAPP	0.421 *	Verified	1.623	0.293		
H8	PR	→	WAPP	0.399 *	Verified	3.584	0.278		
H9	LTPW	→	WAPP	−0.652 ***	Verified	2.497	0.454		

Notes: PC: path coefficient. * *p* < 0.05, ** *p* < 0.05, *** *p* < 0.01, VIF: variance inflation factor.

## Data Availability

The data will be made available on reasonable request from the corresponding author.

## Supplementary Materials

The following are available online at https://www.mdpi.com/article/10.3390/ijerph18116167/s1, S1: Informed Consent Form.

## Appendix A

**Table A1:** Expert participants engaged in the assessment and testing of the questionnaire.

Sr.	Profession	Institution	Experience (Years)	Communication
1	Professors, Associate professors (Sociology, Medical, Psychology)	QAU, KEC, FCCU	10–30	Email/Telephone
2	Medical practitioners	SIH, PIMS, AKUH	10–15	Email/Telephone
3	Healthcare counselor and advisor	NIH	More than 20	Email

Notes: QAU: Quaid-i-Azam University, KEC: King Edward College, FCCU: Forman Christian College University, SIH: Shifa International Hospital, PIMS: Pakistan Institute of Medical Sciences, AKUH: Aga Khan University Hospital, NIH: National Institute of Health.

## Appendix B

**Table A2:** List of questions included in the questionnaire survey conducted.

Constructs	Items	Degree of Agreement				
		5	4	3	2	1
Mythical attitude towards pandemic (MAP)	MAP_1_: I think the adoption of preventive measures will not be helpful in pandemic containment.					
	MAP_2_: I think this pandemic (COVID-19) will vanish on its own.					
	MAP_3_: I think adopting preventive measures cannot keep me healthy.					
	MAP_4_: I think the adoption of preventive measures is useless for me because I need to go out to earn a livelihood.					
	MAP_5_: I think COVID-19 will automatically die due to high temperatures.					
Pandemic knowledge (PK)	PK_1_: The COVID-19 may transmit through human-to-human interaction.					
	PK_2_: The COVID-19 may also transmit through a common point of contact (door, table surface, etc.).					
	PK_3_: The COVID-19 may transmit through handshake and communication with the carrier of this disease.					
	PK_4_: The initial symptoms of COVID-19 include fever, dry cough, sneezing, body aches, and breathing distress, etc.					
	PK_5_: The infectious diseases may be prevented if we keep ourselves clean.					
	PK_6_: Disease (COVID-19) can be prevented through continual handwashing.					
	PK_7_: The COVID-19 enters the human body through the nasal (nose) and oral (mouth) cavity as well as the eyes.					
	PK_8_: The COVID-19 can be prevented through social distancing.					
Ease of pandemic prevention adoption (EPPA)	EPPA_1_: I think face masks would be sufficient if there is a long-term outbreak.					
	EPPA_2_: I think home quarantine would be feasible if there is a long-term outbreak.					
	EPPA_3_: I think the food supplies would be sufficient if there is a long-term outbreak.					
	EPPA_4_: There is a sufficient amount of disinfectants, soaps, and hand sanitizers for the long-term outbreak.					
Self-efficacy (SEF)	SEF_1_: I have the prevention instructions for the pandemic (COVID-19).					
	SEF_2_: I have the required capital (face masks, sanitizers, and disinfectants, gloves) to take preventive measures.					
	SEF_3_: I have the skills to adopt preventive measures.					
	SEF_4_: I can completely adopt the preventive measures.					
	SEF_5_: I believe I will adopt these measures until the outbreak persists.					
Peer groups’ beliefs (PGB)	PGB_1_: I am adopting pandemic preventive measures because my peer groups (friends, colleagues, family physicians, and health professionals) are doing so.					
	PGB_2_: I am adopting preventive measures as they are suggested by my family physician.					
	PGB_3_: I am adopting preventive measures as they are suggested by health professionals.					
	PGB_4_: I am adopting preventive measures as they are suggested by my colleagues, friends, and neighbors.					
Moral values (MV)	MV_1_: I am morally responsible for preventing others from being infected because of me (if I am infected).					
	MV_2_: It is my moral obligation to provide supplies of masks and disinfectants to others if I have their excess supply.					
	MV_3_: It is my moral obligation to reduce the usage of masks and disinfectants to spare them for others.					
	MV_4_: If I have any symptoms (fever, dry cough, etc.) I am responsible for informing the relevant health authorities.					
	MV_5_: I am responsible for adopting preventive measures not only for myself but also for others.					
Risk-averse behavior (RAB)	RAB_1_: I am adopting preventive measures to keep myself healthy.					
	RAB_2_: I am adopting preventive measures to keep my kids/parents/siblings/spouse healthy.					
	RAB_3_: I am advising my kids/parents/siblings/spouse to adopt preventive measures.					
	RAB_4_: I am avoiding visits to crowded places and staying at home most of the time to avoid contact with strangers.					
	RAB_5_: I am practicing social distancing to prevent COVID-19.					
Perceived risk (PR)	PR_1_: I perceive the severity of the disease (COVID-19).					
	PR_2_: I understand the susceptibility of the health risk of this disease (COVID-19).					
	PR_3_: I think this (COVID-19) is a fatal disease.					
	PR_4_: This disease (COVID-19) does not discriminate against gender, race, ethnic groups, countries, and borders.					
	PR_5_: The outbreak may persist if people are not quarantined.					
Lack of trust in political will (LTPW)	LTPW_1_: The government does not respond timely to the economic problems.					
	LTPW_2_: It is not in the interest of the government to prevent people from diseases.					
	LTPW_3_: Government is not willing to provide better health facilities to the people.					
	LTPW_4_: The government is not doing enough for the people who got unemployed during the pandemic outbreak.					
	LTPW_5_: It is not in the interest of the government to follow transparency.					
Willingness to adopt pandemic prevention (WAPP)	WAPP_1_: I intend to adopt preventive measures if any outbreak happens in the future.					
	WAPP_2_: I am ready to be quarantined to prevent the outbreak of the pandemic (COVID-19).					
	WAPP_3_: I intend to highly recommend the preventive measures to others.					
	WAPP_4_: I have the intention to adopt a healthy lifestyle even after the outbreak.					
	WAPP_5_: I intend to adopt preventive measures during the present outbreak of COVID-19.					
	WAPP_6:_ If there is a long-term outbreak, I would be willing to be home quarantined for a long time.					

**Notes:** the degree to agree with the affirmative response is classified as: 5 = “Totally agree”, 4 = “Agree”, 3 = “Neutral”, 2 = “Disagree”, 1 = “Totally disagree.”
