# Supplementary material for: Intention-Based Critical Factors Affecting Willingness to Adopt Novel Coronavirus Prevention in Pakistan: Implications for Future Pandemics
Source: Int J Environ Res Public Health. 2021 Jun 7;18(11):6167. doi: 10.3390/ijerph18116167 (PMC8200947; doi:10.3390/ijerph18116167)
Supplement: Supplementary file 1 [file ijerph-18-06167-s001.zip › ijerph-1219526-supplementary.pdf]

Informed Consent Form

This informed consent form is for the local residents whom we are inviting to participate in the research, entitled **"Intention-Based Critical Factors Affecting Willingness to Adopt Novel Coronavirus Prevention in Pakistan: Implications for Future Pandemics".**

This Informed Consent Form has two parts:

Part I: Information Sheet

I am Dr. Munir Ahmad (the principal investigator), working for Zhejiang University. I am doing research on the COVID-19 which is very common in this country now-a-days. I am going to give you information and invite you to be part of this research. This consent form may contain words that you do not understand. Please ask me to stop as we go through the information and I will take time to explain. If you have questions later, you can ask them of me.

The confirmed COVID-19 cases are increasing at an alarming rate in your community. We want to assess public behavior and willingness to adopt pandemic prevention to stop the current pandemic. We believe that you can help us by telling us what you know both about pandemic and about local health practices for the containment of this pandemic. We want to learn what people who live or work here know about the causes of COVID-19 and why some people get it. We want to learn about the different factors that motivate or inhibit residents from adopting pandemic prevention. We also want to know more about local health practices because this knowledge might help us to learn how to better control the pandemic in this community.

You are being invited to take part in this research through your participation in a questionnaire form because we feel that your presence as a society member can contribute much to our understanding and knowledge of local health practices.

The information recorded is confidential, you do not need to fill in your name. The filling of questionnaires is totally a volunteer process. There will be no direct benefit to you, but your participation is likely to help us find out more about the factors affecting public willingness regarding pandemic prevention adoption.

You can ask any question about any part of the research study, if you wish to.

Thank you very much!

Part II: Certificate of Consent

(This section is mandatory)

I have read the foregoing information. I have had the opportunity to ask questions about it and any questions I have been asked, have been answered to my satisfaction. I consent voluntarily to be a participant in this study.

Print Name of Participant__________________

Signature of Participant ___________________

Date ___________________________

Statement by the researcher taking consent

I have accurately read out the information sheet to the potential participant. I confirm that the participant was given an opportunity to ask questions about the study, and all the questions asked by the participant have been answered correctly and to the best of my ability. I confirm that the individual has not been coerced into giving consent, and the consent has been given freely and voluntarily.

A copy of this ICF has been provided to the participant.

Print Name of Researcher taking the consent________________________

Signature of Researcher taking the consent__________________________

Date ___________________________
